# Physical Education: The Effect of Epoch Lengths on Children’s Physical Activity in a Structured Context

**DOI:** 10.1371/journal.pone.0121238

**Published:** 2015-04-13

**Authors:** Alberto Aibar, Julien Chanal

**Affiliations:** 1 University of Geneva, Geneva, Switzerland; 2 Distance Learning University, Brig, Switzerland; Karolinska Institutet, SWEDEN

## Abstract

**Background:**

Despite a consensus emerging that affirms that shorter epochs should be used in youth to correctly register physical activity levels in free-living conditions, little is known about its effect on children’s physical activity conducted in structured periods of time. This study analyzed the effect that epoch length (1, 2, 3, 5, 10, 15, 30 and 60s) may have on different physical activity intensities in physical education lessons.

**Methods:**

A sample of 1912 individual measures of physical education lessons were measured with a GT3X accelerometer. Data were collected from 1227 Swiss Elementary school students recruited in 17 elementary schools. PE lessons lasted from 45 minutes to one and a half hours. Data, originally collected in 1-s epoch, were then reintegrated into 2s, – 3s – 5s – 10s – 15s – 30s –60s epochs.

**Results:**

Longer epochs were associated with higher levels of light (F = 8197.6, p < .001), moderate (F = 2708.17, p < .001), and moderate-to-vigorous physical activity (F = 888.08, p < .001). However, longer epochs showed lower levels of sedentary activity (F = 31714.33, p < .001) and vigorous physical activity (F = 1910.97, p < .001). Bias increased in all PA intensities when shorter epochs were compared with longer epochs. There were statistically significant differences in compliance with physical education guidelines (χ2 = 989.27, p<.001), showing higher levels with longer epochs.

**Conclusion:**

PA context may have some influence on the effects that epoch length have on PA estimates, more specifically on MVPA. Nevertheless, the use of a high-frequency sampling interval should be used to more accurately assess children’s PA.

## Introduction

Accurately measuring physical activity (PA) levels is absolutely necessary in order to conduct meaningful health research. Accelerometers have emerged as one of the most used tools to provide an objective, practical, accurate and reliable measure of PA [1]. Accelerometers become very useful tools, especially in some populations, such as children or adolescents, where other measurement methods (e.g., questionnaires) may be much less accurate [2] due to the nature of their PA practice. Empirical data have provided evidence that shows that PA among youth is usually sporadic and intermittent [3–5]. Therefore, the use of accelerometers may make it easier for researchers to capture youth PA. However, several methodological decisions must previously be made in order to obtain the most accurate measurement, such as the sampling interval [6–7].

A consensus is emerging that shorter epochs should be used in youth to correctly register PA levels in free-living conditions [8]. Given that moderate-to-vigorous PA (MVPA) is an essential variable in terms of health, several recent studies [7, 9–10] have focused on the importance of the sampling interval in this intensity, showing in general terms how MVPA scores seem to decrease as the epoch lengths increase [2, 11–12]. Due to the intermittent and sporadic PA in youth [4], this fact suggests that PA may be better measured with shorter epochs. However, studies are usually conducted in free-living conditions [6, 13], so further development and refinement of methodological decisions related to epoch length are required [14].

Despite the fact that it is generally assumed that shorter epochs should be used instead of longer epochs to enhance quality of PA level estimation, a recent study conducted with adolescents [8] has suggested that the PA context can influence the effect of variations in epoch length on PA estimates. Few studies [15] have been carried out on children in order to analyze the effect of epoch length on PE lessons. To our knowledge, the research conducted by Sanders and colleagues [8] has been the first study on this topic that has analyzed the sampling interval effect within a structured context such as the physical education (PE) lesson, on a large sample. In further depth, it showed that estimates of MVPA increased as epoch increased from 1-s to 60-s, having the inverse relationship to the usual effect in free-living conditions [7, 9, 13, 16]. McClain and colleagues [15] also did a methodological comparison using cut-points that do not correspond to the latest recommendations [17]. Given that PE plays a key role in the transformation of behaviors and the adoption of healthier lifestyles [18], further and more in-depth analyses of this structured period in terms of children’s PA practice are fully warranted.

School is a particularly outstanding environment for providing and promoting MVPA [19], because it is the only setting that reaches nearly all children, most of whom spend almost half of their waking day at school. PE lessons have been established as the only period where teacher can provide MVPA to virtually all students [20]. The recommended amount of MVPA has been operationalized as students spending 50% of their PE time being physically active [20]. From a practical point of view, measurement of students’ PA in PE lessons usually amounts to a simple percentage of MVPA out of the total PE time. This fact allows us to categorize children as active or not active. As other studies have demonstrated [9, 21], sampling intervals seem to affect compliance with daily guidelines (i.e., 60 min/day of MVPA) in free-living conditions. Nevertheless, no study has analyzed the effect of epoch length on compliance with guidelines in a structured period of time such as a PE lesson. Consequently, further research is required in order to avoid any mistakes in the final epidemiological conclusions of PE research.

The main objective of this study was to study and analyze the effect that epoch length (1, 2, 3, 5, 10, 15, 30 and 60s) may have on different PA intensities, specifically in a structured time period in terms of PA practice, such as PE lessons. The number of studies that are interested in the hypothetic effect of the epoch time have increased due to improvements in accelerometer data storage capability, so further research may be conducted to address this question in depth [22] and see what the possible consequences of methodological decisions are in other PA contexts. In addition, several secondary objectives were established. Firstly, we tried to test the effect of epoch length on compliance with PE guidelines in relation to MVPA practice. Secondly, an analysis was conducted as to whether the length of a structured period of time may have some influence on the effect of epoch length.

## Materials and Methods

### Study sample and design

Data were collected from 1227 Swiss elementary school students (5th, 6th and 7th grade; 52.3% of boys) recruited in 17 elementary schools in the Geneva canton (Switzerland). Schools were selected on the basis of accessibility and willingness to cooperate. As a general rule, each participant was monitored continuously during at least one PE lesson. Nevertheless, there were some of them who were monitored twice throughout the academic year, given that they were included in another broader research project. This fact does not incorporate error into the analysis conducted given that our objective is not to analyze inter-subject differences. All individual measures of PE lessons are treated as independent measures in order to increase sample size and to strength this manuscript. All measurements defined a total final sample of 1912 individual measures of PE lessons. PE lessons are usually organized around a 45-minute period. However, some schools combined two of these periods in just one PE lesson due to merely organizational reasons. These periods could results in lessons of up to one hour and 30 minutes. Consequently, we differentiated between two types of lessons, short lessons (< = 45 minutes; n = 990) and long lessons (>45 minutes; n = 922). This study was approved by the research ethics committee from the psychology section of the Psychology and Education Sciences Faculty of Geneva. Written informed consent was obtained from their parents.

### Instruments and procedures

#### Objective Physical Activity

The tri-axial GT3X accelerometer (Actigraph, Pensacola, Florida, USA) was used to continuously assess PA during the PE lessons. The GT3X was attached to an adjustable elastic belt with snap buckles and worn in line with the right hip in accordance with the guidelines suggested [23]. All participants were informed about how to use the accelerometer during the PE lessons. Accelerometers were initialized as described by the manufacturer, and the epoch was set at 1 second.

#### Procedures

Six research assistants were trained to collect data at the different schools. Students were equipped with accelerometers immediately before the PE lesson and data collection started when the PE teacher started the lesson. Lesson time was directly registered by the observer on standardized sheets during each assessed lesson. After registering the PE lessons, the accelerometers were downloaded in order to obtain raw file data.

File data, initially registered at 1-s epoch, were reintegrated into 2s – 3s – 5s – 10s – 15s – 30s –60s. The 1s- and 60s- epoch were established as the limit values of a normal range of epoch lengths. A broad range of epoch lengths was established in order to perform a richer methodological comparison. Moreover, these lengths were chosen due to their common use in literature in order to clarify their most appropriate use.

The output obtained (counts.min^-1^) was converted into time (min/day) spent in different PA intensities. To do that, and based on a recent review 17, Evenson cut-points [24] were applied to vertical axis data in order to calculate time spent in different PA intensities. More specifically, cut-off points (15 s -1) were <25 counts for sedentary activity (SA), >26 and <573 for light physical activity (LPA), >574 and <1002 for moderate physical activity (MPA), and ≥1003 for vigorous physical activity (VPA). Cut-off point values were either divided or multiplied for data reintegrated into 1s, 2s, 3s, 5s, 10s, 30s, 45s, and 60s and consequently used to calculate time spent in the different intensities. Time spent in MVPA was determined by adding minutes in a day of MPA and VPA. Compliance with MVPA guidelines during the PE lessons were calculated by considering 50% of the total wear time doing MVPA. Average wear time during all lessons was 51.59 ± 19.98 minutes. It should be also pointed out that average wear time for short lessons was 35.71 ± 5.28 minutes whereas for long lessons it was 67.87 ± 13.57 minutes. Due to differences in wear time between lessons, all statistical analyses were conducted with variables of the different PA intensities expressed in percentages of total wear time.

### Statistical analysis

Means and standard deviations of different PA intensities were calculated for the following epoch lengths (1-, 2-, 3-, 5-, 10-, 15-, 30-, and 60-s). These descriptive statistics were also calculated by the category of the lessons in terms of length. One repeated measures ANOVA method was used to analyze the differences between epochs for all PA intensities. Later, Tukey’s post hoc analyses were conducted after any significant ANOVAs to identify specific epoch differences. It should be noted that if Mauchley’s test revealed that the assumption of sphericity had been violated (P < 0.05), the Greenhouse-Geisser procedure was applied to adjust degrees of freedom. The eta-square was used to determine the effect size. Agreement between different epoch lengths for different PA intensities was studied according to the Bland-Altman method [25]. Additionally, charts were designed to show differences between short and long lessons at the different PA intensities. Finally, percentages of adolescents who met PE guidelines (50% of the wear time on MVPA) were calculated. Cochran’s Q test was carried out to analyze if there were significant differences in percentages of compliance with both guidelines between different epochs. The criterion for significance was set at p < 0.05. All statistical analyses were performed using SPSS Statistics 19.0 (Statistical Package for the Social Sciences for Windows, 19.0., 2006, SPSS Inc., Chicago, IL).

## Results

**Fig 1:**
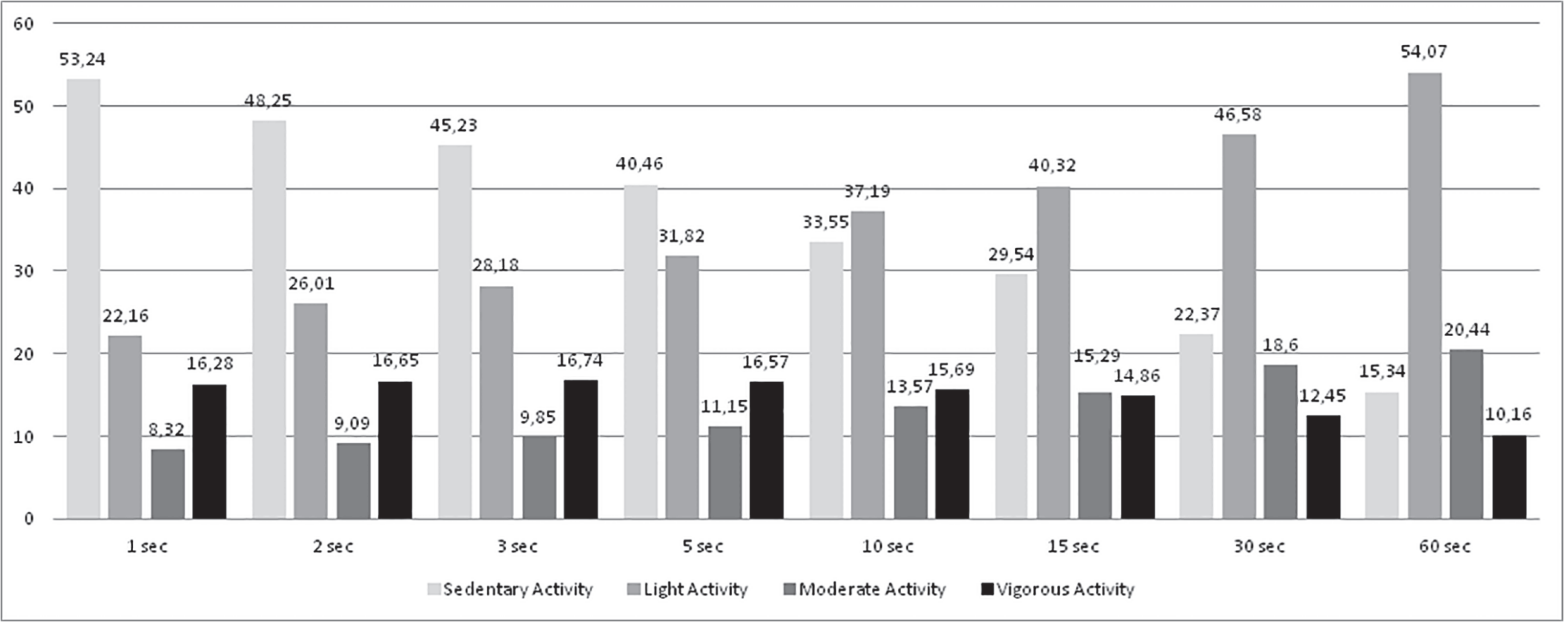
Percentages of sedentary, light, moderate and vigorous physical activity by epoch length for all PE lessons.

Percentages of sedentary, light, moderate and vigorous PA for each epoch length data are shown in Fig 1. As seen in Fig 1, longer epochs showed higher levels of light and moderate activity. On the contrary, this relationship tends to be negative in sedentary and vigorous activity, showing lower levels of SA and VPA when longer epochs were used. The repeated measures ANOVA showed statistically significant effects for SA (F (1.83, 3504.77) = 31714.33, p <. 001; η^2^ =. 943), LPA (F (1.42, 2715.53) = 8197.6, p <. 001; η^2^ =. 811), MPA (F (1.79, 3424.51) = 2708.17, p <. 001; η^2^ =. 586), and VPA (F (2.1, 4013.1) = 1910.97, p <. 001; η^2^ =. 500). Despite the fact that MVPA data are not expressed in Fig 1, one repeated measures ANOVA was also calculated at this PA intensity and a statistically significant effect was also obtained (F (1.51, 2.889.43) = 888.08, p <. 001; η^2^ =. 317). Different percentages were found that increased throughout the epoch lengths, ranging from 24.6% at 1-s epoch to 30.6% at 60-s epoch. Post Hoc Bonferroni revealed that all epoch lengths significantly differed from each other (p <. 001) for all PA intensities. There was only one exception which was between 2-s and 5-sec in VPA.

**Fig 2:**
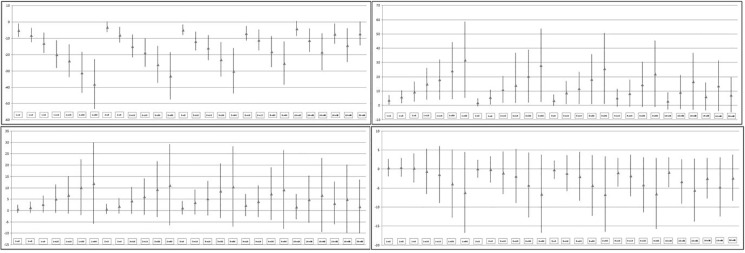
Bias and 95% confidence intervals for each epoch for Sedentary, Light, Moderate and Vigorous Activity.

The mean bias and 95% limits of agreement for all PA intensities obtained from the Bland-Altman analysis are shown in Fig 2A, 2B, 2C and 2D. It could be said that mean biases were rather low for MPA and VPA. However, mean biases in SA and LPA were much longer in comparison with the other PA intensities. Whereas LPA and MPA showed positive biases, SA and VPA showed negative ones. Bias increased in all PA intensities, both in a positive and in a negative way, respectively, when it compared shorter epochs with longer epochs.

As far as the limits of agreement are concerned, the largest were found in LPA (see Fig 2B). The smallest limits of agreement were found in VPA (see Fig 2D). Nevertheless, in all PA intensities, the more distant the compared epochs, the higher limits of agreement were found (e.g., 1-s, 2-s or 3-s versus 30-s and 60-s). In MPA and VPA, small limits of agreement could be found between shorter epochs (e.g., comparison between 1-s and 5-s).

The percentages of children who meet PE guidelines (50% of the total wear time in MVPA) are shown in Table 1. These percentages are also shown by the length of the PE lesson. The longer epoch length used, the higher the percentage of compliance was found in the 50% guideline. This tendency was also observed in short as well as in long PE lessons. Nevertheless, percentages of compliance are basically higher in short PE lessons than in longer ones, showing how an incrementally positive tendency is reached when the epoch increases. Cochran’s Q Test was applied in order to test differences between epoch lengths in compliance with guidelines. There were statistically significant differences in the 50% guideline (χ2 = 989.27, p<.001). These differences were also found both in short PE lessons (χ2 = 561.35, p<.001) and in long PE lessons (χ2 = 433.97, p<.001). It can also be appreciated how percentages of compliance are low, going from 0.1% to ~11%.

**Table 1:** Percentages of compliance with guidelines (50% of wear time in MVPA) for PE lessons.

	1-sec	2-sec	3-sec	5-sec	10-sec	15-sec	30-sec	60-sec
All PE lessons	0.1	0.4	0.6	1	2.8	4	8.2	11.5
Short PE lessons	0.1	0.5	0.7	1	3.7	5.1	9.3	12.7
Long PE lessons	0.1	0.2	0.5	1.1	1.8	2.8	7	10.2

**Fig 3:**
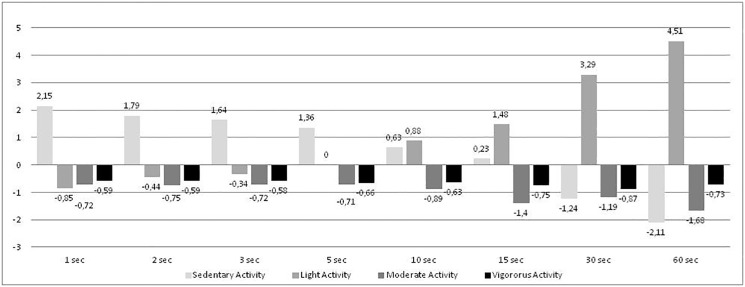
Differences between short and long PE lessons by epoch for each PA intensity. Note: Values are expressed in minutes. Values of long lessons have been subtracted from short lessons.

Fig 3 shows the differences between short and long PE lessons for each epoch length. MPA and VPA showed a regular tendency in their differences, not increasing or decreasing based on the epoch length. However, differences in SA and LPA showed a tendency that respectively decreases and increases with longer epoch lengths. For SA and LPA, there is a turning point between 5-s and 10-s epoch where the sign of the difference between short and long PE lessons changes direction.

## Discussion

The main objective of this study was to study and analyze the effect that epoch length (1, 2, 3, 5, 10, 15, 30 and 60s) may have on different PA intensities, specifically in a structured time period, such as PE lessons. Findings showed a statistically significant effect of epoch length in all PA intensities measured. However, directions of the effects were different among PA intensities. Longer epochs were associated with higher estimations of MVPA, MPA and LPA. On the contrary, VPA and SA were inversely associated, showing a negative relationship with epoch length. We will discuss these results in greater depth from here onwards.

Considering all PA intensities, our results agree with the findings of Sanders and colleagues in the PE context [8]. Increases in estimates of MVPA, MPA and LPA are likely to result in VPA being misclassified as lower intensity. Moreover, increases in LPA could also be the result of misclassification of SA as higher intensity. These over- and underestimations seem to result from the changes in the distribution of PA among the different intensities when longer epoch lengths are used. Other studies have already shown that longer epochs might underestimate VPA levels in young people [7, 11]. Along the same line, other studies have also shown that SA levels seem to decrease when longer epochs are used, both in free-living conditions [16, 21] and structured time periods [8]. Considering the mean biases and the limits of agreement of the different PA intensities in our study, findings lead us to agree with the recommendation about the use of shorter epochs in order to avoid error of measurement, especially for high PA intensities. In addition, the levels of agreement observed between shorter epochs (from 1-s to 5-s) seem to suggest that studies conducted with these epochs could be compared in order to obtain final conclusions.

From a practical research perspective, MVPA is one of the most commonly used variables in health research to determine the active or inactive character of the population. Given that high PA intensities are usually underestimated, Reilly and colleagues [2] proposed a practical solution classifying moderate and vigorous PA together, as MVPA, in order to provide a more useful outcome in youth health research. However, significant differences in MVPA can be also extensively found in international literature [6, 9]. In our study, the higher estimations of MVPA found with longer epochs is an important finding, which seems to agree with other previous studies conducted in structured time periods [8, 15] and free-living conditions in children [7]. However, the direction of the relationship is inversely associated with the majority of studies carried out in free-living conditions [10–12]. This inverse relationship leads us to affirm that the PA context may have some influence on the effects that epoch length has on PA estimates, more specifically on MVPA. Further research would be warranted to study other structured time periods such as recess, trainings, etc.

In a recent research study, Sanders and colleagues [8] have found that the association of MVPA with epoch length differs between the PE context and the leisure time context in the same sample. Given that our findings show the same tendency in comparison with other studies of children under free-living conditions [10, 12], we propose a hypothesis that could explain this difference in tendency in PE lessons. The PE lesson is a period of time that is usually totally organized by the PE teacher. A normal PE lesson combines periods of PA practice with moments of organization and explanation of these activities. Since PA periods are established by the PE teacher from start to finish, PA, during those PA periods, may have been basically organized in bouts of PA, in other words, the PA was carried out in a more continuous way. The effect of epoch length could be influenced by this fact, as we can deduct from the conclusions of other studies. For instance, it has been shown recently [13] that time spent on bouts of MVPA increases with longer epochs. It is likely that structured time periods, such as PE lessons, are basically comprised of bouts of PA, which could explain the same tendency in the epoch length effect. Further research in order to study this hypothesis in greater depth should be conducted in future studies.

Several secondary objectives were also established at the beginning. Firstly, we tried to test the effect of epoch length on compliance with the PE guideline, 50% of the wear time doing MVPA. It can be seen that percentages of compliance with guidelines were significantly different depending on the epoch lengths used. These findings agree with other studies conducted in children [12] and in adolescents [9], but in free-living conditions. To our knowledge, no studies have analyzed this phenomenon in PE lessons. However, due to the characteristics of epidemiological research, it could be affirmed that there is an important risk of misinterpretation based on epoch length used. Consequently, even in a structured time period, epoch length should be considered and coherently programmed to prevent this undesirable effect. Short PE lessons showed higher percentages of compliance than long PE lessons. This element could be explained by the fact that short periods of time include fewer factors that disturb the PE activities, such as fatigue, explanations or change of activities, and therefore the engagement in PA is greater in terms of percentage. By analyzing the effectiveness of PE lessons in terms of MVPA practice [20], data show how only a small percentage of children comply with guidelines. Nevertheless, these negative results, from the epidemiological point of view, could be treated either from a catastrophic and apocalyptic perspective or simply as a dramatic situation. Unfortunately, the correct use of the epoch length would contribute to generating terrible conclusions in terms of PA practice.

Secondly, we proposed to analyze if the length of a structured period of time could have some influence on the epoch length effect. We hypothesized at the beginning that duration of PE lessons would not have any influence on the epoch length effect. Neither short nor long periods of time should affect the impact that the sampling interval has on the quantification of different PA. We were able to observe both in short PE lessons and in long PE lessons that the effect of the epoch length on different PA intensities was similar. Nevertheless, we could appreciate (see Fig 3) that the duration of the period of time registered could enhance or diminish the effect for some PA intensities. Despite the fact that MPA and VPA seem not to be modified based on the duration of registered time, SA and LPA showed a change in the effect from 5-s epoch. While SA decreases in long PE lessons for long epochs, LPA seems to incorporate that activity. Nevertheless, scores of MVPA, which is the most common intensity for epidemiological conclusions, were not modified either, depending on the duration of registered time. Therefore, we could conclude that short epoch lengths (1-, 2-, 3-, and 5-s) seem not to be affected by the duration of the registered period of time, so, once again, we recommend their use in order to minimize error of measurement as much as possible.

Limitations and strengths of this study should be noted. An obvious limitation was the lack of a criterion measure, such as direct observation, thus avoiding comparing and determining which epoch length is actually the most accurate estimate of PA in children. In addition, we did not analyze bouts of PA and how these may change based on the epoch length effect. This is an important limitation especially when it is known that accrediting PA in bouts may have health benefits beyond the total amount of PA done [26]. Moreover, analyzing bouts of PA depending on the length of the PE lessons would be really interesting as bouts should be much related to this type of structured time period. Further research is needed in future analyses on this topic. Finally, the recommended Evenson’s cut-point [17], which has been used in this study, was not specifically created for all epoch lengths used, but only for 15.s epoch. However, it does not make any sense to create a new set of cut-points for every epoch as this would cause further misunderstanding in data comparison [27]. This fact leads us to affirm that the use of Evenson’s cut-point is an important strength of this research. Another strength consists of the large amount of PE lessons that were analyzed. Moreover, activities or sport that comprised the PE lessons were highly varied and therefore conclusions may be more trustworthy. Finally, assessment of compliance with guidelines according to epoch lengths has been conducted in children during their PE lessons for the first time to the best of our knowledge.

In summary, our findings seem to corroborate the current suggestion that the use of a high-frequency sampling interval should be used to more accurately assess children’s PA [8, 15]. Estimated MPA and LPA increased with epoch length in PE lessons, while VPA and SA decreased. Estimated MVPA and, consequently, percentages of compliance with PE guidelines increase when an epoch length increases as well. Findings related to MVPA show an inverse relationship in comparison with data found in the literature in free-living conditions. This fact seems to suggest that the context where PA occurs may influence the effect of epoch length on PA estimates. Specifically, structured time contexts, in which a high density of PA bouts is more likely to be found, may change the direction of the epoch length effect. Due to their high conformity, epidemiological studies should be carried out with shorter epochs if researchers want to compare international studies.

## Perspectives

Our findings highlight that there is a statistically significant epoch length effect in all PA intensities. This study has shown that estimated MVPA—the PA intensity usually selected as reference to compliance with guidelines—shows higher values when epoch length increases. This association within the PE lesson context is inversely related to the normal one that can be seen in free-living conditions. This fact leads us to conclude that, on one hand, epidemiological studies should consequently consider the sampling interval to offer correct conclusions about percentages of children who meet compliance with PE guidelines. On the other hand, these findings corroborate the fact that the context within which PA is measured may reduce the estimate of PA. Consequently, comparisons should not be made between studies using short (e.g., 1, 2, 3, 5-s) and long epochs (e.g., 30 and 60-s). In addition, the length of the time period registered may also be especially influenced by the epoch length effect for long sampling intervals. In conclusion, shorter epochs (e.g., 1, 2, 3, 5-s) may be the most appropriate in all PA contexts.

## Supporting Information

S1 Dataset Dataset file. (SAV)
